# A Gamified Pain Management Intervention for Adults With Chronic Pain in Mainland China: Single-Arm Pre-Post Pilot Study With Machine Learning Predictive Modeling

**DOI:** 10.2196/78823

**Published:** 2026-04-07

**Authors:** Mun Yee Mimi Tse, Jiafan He, Tyrone Tai On Kwok

**Affiliations:** 1 School of Nursing and Health Sciences, Hong Kong Metropolitan University, 1 Sheung Shing Street, Homantin, Kowloon, 999007, China (Hong Kong), 852 39708764; 2 The Jockey Club Institute of Healthcare (IOH), Hong Kong Metropolitan University, Kowloon, China (Hong Kong)

**Keywords:** gamification, artificial intelligence, machine learning, pain management, chronic pain

## Abstract

**Background:**

The widespread prevalence of chronic pain (CP) significantly impacts daily functioning worldwide. In mainland China, maintaining engagement in biopsychosocial interventions remains challenging. Gamification, designed based on self-determination theory, can enhance motivation, while machine learning (ML) algorithms can assist clinicians in dynamically optimizing pain management.

**Objective:**

This study aimed to (1) evaluate the preliminary effectiveness of a gamified pain management (GPM) program on CP and psychological outcomes and (2) identify key factors of significant pain improvements through the application of ML to guide intervention adjustments.

**Methods:**

A single-arm, pre-post study was conducted with 16 participants with CP in mainland China, recruited via social media using convenience sampling. Participants engaged in a 10-week web-based GPM intervention consisting of education, physical activities, and gamified elements, including points, avatars, and feedback. Primary outcomes were pain intensity and interference measured by the Brief Pain Inventory. Secondary outcomes included anxiety, depression, and quality of life. Analysis included paired *t* tests, and ML models were trained to predict clinically meaningful pain reductions. Shapley additive explanations, least absolute shrinkage and selection operator regression, association rule mining, and Kaplan-Meier survival analysis were used to identify key predictors and optimal sessions and intervention durations across subgroups.

**Results:**

A total of 16 participants were engaged, with a mean age of 27.63 (SD 9.584) years. Results from paired *t* tests reported significant improvements in pain intensity (decreased by 27.3%, 95% CI 1.061 to 3.064; *P*=.001), pain interference (decreased by 27.3%, 95% CI 8.159-17.216; *P*<.001), and psychological distress, including anxiety (*t*_15_=3.538, 95% CI 0.969 to 3.906; *P*=.003) and depression (*t*_15_=4.559, 95% CI 2.230 to 6.145; *P*<.001). The gradient boosting model demonstrated the highest predictive accuracy (area under the curve=0.89 and accuracy=0.82). Least absolute shrinkage and selection operator regression identified session 3 (β=−0.45, 95% CI −0.68 to −0.22; *P*<.001) and session 5 (β=−0.32, 95% CI −0.59 to −0.05; *P*=.02) as most predictive of clinical success, while association rule mining revealed effective session combinations for different patient subgroups. Time-to-event analyses indicated that individuals with low back pain and higher baseline severity required longer intervention durations for improvement (5 wk; *P*=.03).

**Conclusions:**

This pilot study presents an innovative method that combines ML with dynamic engagement data from a GPM program during interventions, rather than relying on static baseline data in prior studies. The results show preliminary efficacy and identify specific optimal session combinations and personalized treatment durations for different pain subgroups. These exploratory findings contribute to the field by providing a data-driven method for adaptive, personalized digital health interventions that move beyond one-size-fits-all strategies, potentially enabling clinicians to modify content and dosage to improve engagement and outcomes if validated in larger sample trials.

## Introduction

### Problem

The Global Burden of Disease Study reported a high prevalence of chronic pain (CP) globally [1]. CP, defined as pain that persists for more than 3 months, significantly disrupts various aspects of daily life, including work, physical activity, and social engagement. It is frequently comorbid with anxiety and depression, sleep disturbances, and decreased productivity [2]. In mainland China, the prevalence of CP ranges from 50% to 70% and there is a preference for self-management [3,4], highlighting the urgent need for effective pain management. Despite the growing adoption of biopsychosocial approaches in clinical guidelines [5], individuals with CP face strong barriers to adhere to regular physical activity, including fear of pain and lack of personalized plans [6,7].

### Literature Review

To tackle these challenges, gamification has emerged as an innovative strategy to enhance user engagement, motivation, and adherence to health interventions [8]. Drawing on theories such as self-determination theory (SDT), gamification incorporates game design elements into nongame contexts to motivate behavior change [9,10]. While various gamified interventions have been developed to reduce pain symptoms, the treatment effects have been inconsistent. Some studies have shown immediate efficacy for both adults and older adults [11,12], with evidence suggesting that individuals across age groups find these interventions enjoyable and accessible [13]. However, other randomized controlled trials reported nonsignificant effects on pain intensity [14,15]. This heterogeneity may stem from the complex and multidimensional nature of CP, as well as insufficient theoretical grounding and inability to tailor to specific populations [16], a challenge that causes treatments to struggle to maintain adherence over time. Therefore, this study developed a gamified pain management (GPM) program for adults in mainland China, grounded in SDT, to foster intrinsic motivation through multiple game elements such as avatar, choices, feedback, and challenges.

Building on the motivational benefits of gamification, integrating artificial intelligence (AI) can further enhance personalization and long-term adherence through data-driven adaptation by addressing barriers, including accessibility and sustained engagement. Numerous studies have demonstrated that AI can assist in treatment decision-making, dynamic prediction, and behavior guidance. For instance, rule-based recommendation systems can generate personalized exercise plans based on users’ pain characteristics, improving intervention adaptability [17]. AI chatbots provide psychological support and behavioral reminders through real-time interaction, enhancing user engagement and treatment adherence [18]. Machine learning (ML) models including random forests and extreme gradient boosting have been used to predict postoperative pain outcomes and determine opioid use, thereby assisting in drug management [19,20]. These advantages mark a shift from traditional, rule-based systems to dynamic, data-driven prediction models.

Unlike existing studies that primarily rely on static baseline data, our study uses dynamic real-time behavioral data recorded during the intervention engagement into ML models. Moreover, most studies focus on the binary questions of whether intervention reduces pain, but questions such as “how quickly improvement occurs” and “through which mechanisms improvement occurs” remain unexplored, thus lacking a deeper understanding of the time course and underlying processes of intervention effectiveness.

### Aims and Objectives

In response to these gaps, the present pilot study aimed to (1) assess the effects of the GPM intervention on CP in adults in mainland China and (2) identify key factors that predict significant improvements in pain intensity using ML models.

## Methods

### Study Design

This single-arm, nonrandomized, pre-post pilot study aimed to evaluate the preliminary effects of the digital GPM intervention and to develop exploratory ML models predicting clinical pain reduction outcomes. Participants were recruited using convenience sampling through social media platforms and engaged with the intervention via a web-based platform. The study adhered to the TRIPOD-AI (Transparent Reporting of a Multivariable Prediction Model for Individual Prognosis or Diagnosis-AI) reporting guidelines (Checklist 1).

### Ethical Considerations

The work described was carried out in accordance with the guidelines of the Hong Kong Metropolitan University Research Ethics Committee (reference HE-SF2024/34) and was registered on the Chinese Clinical Trial Registry (registration number ChiCTR2400094247). All participants were provided with a detailed digital informed consent form including the study purpose and procedures, potential risks and benefits (no monetary compensation), confidentiality and voluntariness, and contact information for the principal investigator. To ensure privacy and confidentiality, all data were anonymized by assigning a unique reference number, which was also used to log in to the game and surveys. All data are securely stored in an encrypted folder, and only the research team has access to the information. No images containing identifiable participant identities were obtained. No monetary compensation was provided for participation in this study.

### Participants

No formal prior power calculation was performed due to the exploratory, feasibility-testing nature of this pilot study, and a sample size larger than 10 was deemed appropriate for the formative objectives [21]. Interested individuals were recruited through an online flyer via social media platforms including Redbook and WeChat. The flyer featured a QR code to access an online questionnaire for initial eligibility screening. To be eligible to participate in the study, the participants must meet all inclusion criteria: (1) aged 18 years or older; (2) able to read and understand the Mandarin Chinese language; (3) owners of smartphones with internet access; (4) have a previously diagnosed chronic noncancer pain condition lasting more than 3 months; (5) report a score of greater than or equal to 2 on the Visual Analog Scale [22]. Exclusion criteria were as follows: (1) patients with associated pathologies that make it impossible to perform physical exercise (myopathies, neurological diseases, cardiac disease, pregnancy, pulmonary diseases, infection, and fracture) [23]; (2) individuals undergoing surgery or invasive treatments in the last 3 months [24]; (3) concurrent participation (or participation within the preceding 3 mo) in a supervised exercise program or multidisciplinary treatment.

### Intervention

The GPM was open for 10 weeks, allowing participants to complete the web-based intervention at their own pace. It integrated gamification, education, and progressive exercise within a gamified framework informed by SDT. Prior to the first session, participants received web-based instructions outlining the intervention structure. The program was structured as an escape room game, where participants, via hero avatars, progressed through themed sessions by unlocking educational content, exercise tasks, quizzes, and relaxation activities.

The educational modules covered the following information via animated videos and notes: (1) the definition and mechanisms of pain, (2) distinctions between acute and CP, (3) the biopsychosocial impact of CP, and (4) pharmacological and (5) nonpharmacological management strategies [25]. Each progressive exercise module (5‐7 min each) included strengthening and stretching [5] routines tailored to participants’ primary pain locations (eg, neck, shoulder, back, knee, or head) (Table 1). Gamification elements included a points system (awarded for quiz accuracy and exercise completion), a hero avatar, choices and challenges, and positive or corrective feedback mechanisms. To progress, a minimum of 60 points per session was required. Hidden sessions embedded within the game provided opportunities for additional exercises and engagement.

Engagement data, including treatment duration (TD) (first and last login timestamps), quiz accuracy, and session-specific satisfaction scores, were automatically recorded by the GPM platform. Biweekly WeChat reminders were manually sent to promote adherence.

**Table 1.** Descriptive statistics of engagement and feedback metrics (N=16).

Variable	Mean (SD)	Range
Engagement variables		
Treatment duration (wk)	4.38 (2.19)	1‐10
Number of quizzes correctly answered^a^	6.94 (1.53)	3‐8
GUESS^b^	96.25 (12.69)	68‐115
Cumulative session feedback scores^c^		
Understandability	33.94 (5.26)	25‐40
Comprehensibility	33.94 (5.26)	25‐40
Applicability	32.44 (7.56)	15‐40

a Higher quiz accuracy and satisfaction scores indicate greater understanding and engagement.

b GUESS: Game User Experience Satisfaction Scale.

c Scores for understandability, comprehensibility, and applicability represent the cumulative sum of a participant’s ratings across all 8 intervention sessions. Each item was rated from 0 to 5 per session, resulting in a possible cumulative range of 0 to 40 for each item.

### Data Collection

Participants were recruited via social media posters containing a hyperlink to an online eligibility screening questionnaire [26], where detailed study information and consent form were provided. After providing informed consent, screening questions, including pain condition, medical history, and demographic criteria were embedded within the survey. The survey system automatically submitted an incomplete survey if any exclusionary options were selected. Individuals who met the eligibility criteria were invited to voluntarily provide their WeChat ID to receive automatically generated access credentials for the intervention platform.

Baseline assessments (T0) were administered via the survey platform [26] and distributed to eligible participants through WeChat. Upon completion, participants were provided with access credentials to the GPM intervention site [27]. Postintervention assessments (T1) were collected through the same platform immediately after the intervention was completed approximately 10 weeks after baseline.

### Measurements

A list of outcome measures, including instruments and score ranges, is provided in Table 2.

**Table 2.** Summary of demographic, clinical, and intraintervention feature domains used as input variables for machine learning modeling.

Domain	Description
Demographic	Sex (male or female), age
Clinical	
Pain	Pain duration was assessed by demonstrating specific total months of pain experiences (divided into categories: 3 mo, 4 mo, 5 mo, 6‐9 mo, 9-12 mo, and >12 mo) Pain site was provided with different body sites including head, neck, shoulder, low back, and knee
Functionality	Assessed by Brief Pain Inventory considering the total score (score range 0‐70) and subscale scores (score range 0‐10)
Mental health	Assessed by the 7-item General Anxiety Disorder Scale (score range 0‐21) and the 9-item Patient Health Questionnaire (score range 0‐27)
Fear-avoidance and catastrophizing	Evaluated by Fear Avoidance Beliefs Questionnaire (score range 0‐96) and the total score and subscale scores (score range 0‐42) Pain Catastrophizing Scale considering (score range 0‐52) and the total score and subscale scores (score range 0‐24)
Medication consumption	Considers opioid consumption (yes or no)
Quality of life	Evaluated by the 3-level version of EQ-5D^a^ considering the total score (score range 0‐45) and subscale scores (score range 0‐15)
Exercise adherence	Godin Leisure Time Exercise Questionnaire was used (score range 0‐99)
Motivation	Game User Experience Satisfaction Scale considering the total score (score range 0‐24) and subscale scores (score range 0‐24)
Intraintervention variables	
Quiz accuracy	Number of correct answers
Adverse event	Considers adverse events (yes or no)
Intervention duration	The time span from the first to the last day of participation
Session satisfaction score	Summary score of understandability, comprehensibility, and applicability in each session (score range 0‐15).

a EQ-5D: EuroQol 5-dimension.

#### Primary Outcome

Pain intensity and pain interference were assessed using the Brief Pain Inventory, rated on a scale of 0 to 10. Pain severity was calculated based on the past 24 hours and the “current pain.” The pain interference includes the interference degree of pain on general activity, mood, walking, work, social relationships, sleep, and enjoyment of life. The Chinese scale has good reliability with a Cronbach α of 0.89 [28].

#### Secondary Outcomes

Secondary outcomes were as follows:

- Fear Avoidance Beliefs Questionnaire and Pain Catastrophizing Scale: Used to measure pain perception including attitude toward physical activity, work, rumination, magnification, and helplessness related to pain. The Cronbach coefficients of the Chinese version of the Fear Avoidance Beliefs Questionnaire are high, ranging from 0.75 to 0.85 [29], and Pain Catastrophizing Scale is 0.91 [30].
- Godin Leisure Time Exercise Questionnaire: Used to measure exercise adherence, including times of strenuous exercise, moderate exercise, and mild exercise per week via the website. The percentage of exercises completed out of the total number of prescribed exercises is used to measure total exercise adherence. The Cronbach α is 0.64 and the κ index is 0.94 [31].
- Patient Health Questionnaire-9 items and Generalized Anxiety Disorder-7 items: Used to measure depression and anxiety. The Chinese version of the scale has excellent internal reliability; the Cronbach α of the Generalized Anxiety Disorder-7 items is 0.91 [32] and that of the Patient Health Questionnaire-9 items is 0.86 [33].
- The EuroQol 5-D 3-level: Used to assess an individual’s perceptions of quality of life including mobility, self-care, and usual activities. The Chinese version has been treated as the standard in China [34].
- Game User Experience Satisfaction Scale: Used to evaluate overall game satisfaction, with good internal reliability (Cronbach *α*=0.93) [35].
- Intraintervention variables (collected during or immediately after intervention [T1]):
  - Engagement metrics were assessed through session satisfaction scores (SAT) and quiz accuracy, recorded via the GPM platform.
    - Satisfaction scores were derived from the sum of 3 items rated on a 5-point Likert scale following the completion of each session. These items evaluated the following: (1) Understandability: “Are the materials in this session easy for you to understand?” (2) Comprehensibility: “How much do you understand the materials in this session?” (3) Applicability: “To what extent do you think you would apply this session to future self-management?”
    - Quiz accuracy was calculated as the proportion of correctly answered questions in each session’s embedded quiz.
  - TD was automatically calculated as the total number of weeks between a participant’s first and last login on the GPM platform.

### Statistical Analysis

To test aim 1, descriptive statistics for engagement metrics, including TD, quiz scores, and SAT were calculated. Paired *t* tests were conducted to compare differences between the two stages: baseline (T0) and postintervention (T1) across all outcome measures. The normality of difference scores was examined using the Shapiro-Wilk test. In case of violation of the normality assumption, the nonparametric Wilcoxon signed-rank test was applied. In addition, paired *t* tests were conducted within pain subgroups to examine the within-group intervention effects. The pain intensity level was categorized into two groups: mild-to-moderate (scores ranging from 3 to 6) and severe pain group (scores 7 or higher in Brief Pain Inventory) [36]. Given the small sample size and exploratory, hypothesis-generating nature of this study, no multiple comparisons were applied. This decision was made to minimize the risk of type II errors in this initial investigation. A significant level of *P*<.05 was applied. The analyses were performed using SPSS Statistics Version 23.0 (IBM Inc) and Python 3.11.

### ML Analysis

ML modeling was exploratory and served as proof-of-concept analysis. The dataset comprised 53 features, including baseline demographic, clinical, and intraintervention engagement metrics (Table 2). The binary outcome variable, pain intensity, indicates a clinically significant improvement, defined as a ≥30% reduction in pain intensity measured by the BPI [36]. Continuous predictors were *z*-score standardized, and categorical variables were one-hot encoded.

### Predictive Modeling for Pain Reduction

**Figure 1.**
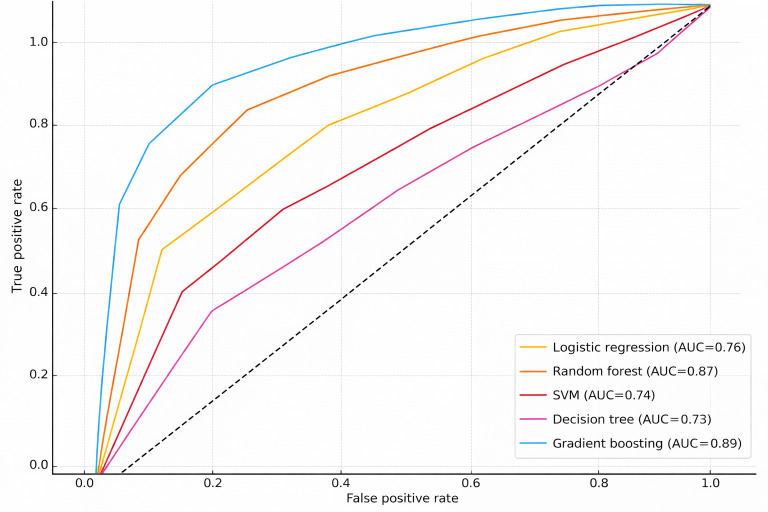
Receiver operating characteristic curve across models for predicting clinically significant pain reduction. AUC: area under the curve; SVM: support vector machine.

Five supervised classification models were trained to predict clinical pain reduction. Due to the limited sample size, model training and hyperparameter tuning used a 5-fold stratified cross-validation procedure on the training data (80% training and 20% testing per iteration) to preserve the distribution of clinical pain reduction across folds. Accuracy, recall, specificity, precision, *F*_1_-score, and the area under the curve (AUC) were used to evaluate performance (Figure 1). To reduce overfitting, model training and evaluation relied on a 5-fold stratified cross-validation procedure, and all models were trained on standardized and encoded features only. Shapley additive explanations (SHAP) was used to identify the most influential predictors of intervention effectiveness from the best performing models (gradient boosting and random forest).

### Session-Level Mechanisms of Clinical Improvement Analysis

A LASSO (least absolute shrinkage and selection operator) regression was performed to further examine each SAT’s contribution to clinical pain reduction. This focused strategy sought to elucidate the role that session-specific engagement played in clinical improvement and to provide guidance for tailoring the order of interventions. A leave-one-out cross-validation approach was used to optimize training data use and guarantee reliable variable selection in the small-sample setting. In order to reduce overfitting, especially in high-dimensional, small-sample situations, this regularized regression technique enables variable selection and coefficient shrinkage. Sessions with the greatest predictive influence were identified by shrinking noninformative variables toward 0.

Association rule mining was then used to find potential interaction patterns for particular subgroups (SAT ≥12 coded as “high”), while LASSO regression identified the contribution of individual sessions. Rules linked to clinical improvement that satisfied high-confidence (≥0.75) and high-lift patterns (≥1.15) thresholds were extracted using the Apriori algorithm. Our threshold of greater than or equal to 1.15 was selected to find patterns where the session combination and clinical improvement co-occurred at least 15% more frequently than would be predicted by chance. Crucially, a lift value greater than or equal to 1 indicates a positive association. The goal of this exploratory analysis was to produce ideas for tailoring the sequencing of digital interventions.

### Time-to-Improvement Analysis by Subgroup

Leave-one-out cross-validation was used with the gradient boosting model to examine the ideal intervention duration needed for clinical improvement across pain subgroups. SHAP-informed patterns in model decision boundaries were used to estimate intervention day thresholds. The robustness and CIs of these thresholds were estimated using bootstrap sampling, which supports reliability under small sample constraints.

The duration of the intervention at which the postintervention assessment satisfied the criteria for clinically significant improvement (≥30% reduction in pain intensity score) was operationalized as the time to improvement. As a result, Kaplan-Meier survival curves were used for time-to-event analysis, with the time to clinical improvement serving as the survival time variable. To investigate variations in median improvement durations by pain site and severity, subgroup comparisons were carried out. Statistical significance was evaluated using log-rank tests. All results should be viewed as preliminary and hypothesis-generating rather than confirmatory, given the small sample size.

## Results

### Participant Demographics

Data collection procedures were completed online between March 2025 and August 2025. A total of 23 individuals accessed the online screening survey. Of these, 5 were excluded for not meeting eligibility criteria (eg, pain duration <3 months, pain intensity <2). Two participants withdrew prior to the intervention due to time conflicts, so no missing data was observed. And 16 participants were enrolled in the study, with a mean age of 27.63 (SD 9.58) years. The majority of participants were employed (15/16, 93.8%), and all had a higher education background. Pain duration varied, with 25% (4/16) of participants experiencing CP for over 1 year, while others reported pain durations between 4 and 12 months. At baseline, participants reported moderate pain intensity levels (mean 5.81, SD 1.38) and pain interference (mean 38.31, SD 12.41) (Table 3).

**Table 3.** Baseline demographics and clinical characteristics (N=16).

Variables	Values
Gender, n (%)	
Male	11 (68.8)
Female	5 (31.2)
Age, mean (SD)	27.63 (9.584)
Marriage, n (%)	
Single	12 (75)
Married	4 (25)
Occupation, n (%)	
Employed	15 (93.8)
Unemployed	1 (6.3)
Education background, n (%)	
Higher education	16 (100)
Duration of symptoms, n (%)	
3 mo	1 (6.3)
4 mo	2 (12.5)
5 mo	3 (18.8)
6‐9 mo	5 (31.3)
9‐12 mo	1 (6.3)
>1 yr	4 (25)
Use of pain medication, n (%)	
Yes	3 (18.8)
No	13 (81.3)
Baseline scores, mean (SD)	
BPI^a^ intensity	5.81 (1.377)
BPI interference	38.31 (12.408)
FABQ^b^	58.69 (13.189)
PCS^c^	35.81 (8.796)
PHQ-9^d^	11.88 (4.674)
GAD-7^e^	9.75 (4.946)
GLTEQ^f^	97.81 (84.680)
EQ-5D-3L^g^	3.06 (2.932)

a BPI: Brief Pain Inventory.

b FABQ: Fear-Avoidance Beliefs Questionnaire.

c PCS: Pain Catastrophizing Scale.

d PHQ-9: Patient Health Questionnaire-9 items.

e GAD-7: Generalized Anxiety Disorder-7 items.

f GLTEQ: Godin Leisure Time Exercise Questionnaire.

g EQ-5D-3L: EuroQol 5-Dimension 3-Level.

### Intervention Effectiveness and Outcome Variables

Table 4 presents the means and SDs of the key outcome measures at baseline and post intervention. Significant reductions were observed in pain intensity (*t*_15_=4.392, 95% CI 1.061-3.064; *P*<.001) and pain interference (*t*_15_=5.971, 95% CI 8.159-17.216; *P*<.001). Secondary outcomes, including pain catastrophizing and psychological distress, also demonstrated significant improvements post intervention. Pain-related catastrophizing (*t*_15_=3.190, 95% CI 2.821-14.179; *P*=.006), depression (*t*_15_=4.559, 95% CI 2.230-6.145; *P*<.001) and anxiety (*t*_15_=3.538, 95% CI 0.969‐3.906; *P*=.003) demonstrated significant improvement. Additionally, quality of life improved significantly (*t*_15_=2.611, 95% CI 0.172-1.703; *P*=.02). Fear-avoidance beliefs and exercise adherence showed no significance (*P*>.05, see Table 4 and Table S1 in Multimedia Appendix 1 for details).

Participants’ engagement with the GPM varied. With a range of 1 to 10 weeks, the average TD was 4.38 (SD 2.19) weeks, demonstrating high flexibility and customized completion rates. The average quiz accuracy was roughly 75%, indicating a generally high level of understanding of the course material. Table 1 displays the cumulative scores for the session feedback items, which were consistently high (mean 33.44, SD 6.03, range 0‐40) throughout all sessions, indicating favorable opinions of the sessions’ comprehensibility and applicability (Tables1  5).

**Table 4.** Preintervention and postintervention comparison of key outcomes (N=16).

Outcomes	Baseline, mean (SD)	Post intervention, mean (SD)	Statistics		
			*t* test *(df)*	95% CI	*P* value^a^
BPI^b^ intensity	5.81 (1.377)	3.75 (1.342)	4.392 (15)	1.061‐3.064	.001
BPI interference	38.31 (12.408)	25.63 (12.387)	5.971 (15)	8.159‐17.216	<.001
PCS^c^	35.81 (8.796)	27.31 (13.200)	3.190 (15)	2.821‐14.179	.006
FABQ^d^	58.69 (13.189)	53.81 (16.586)	1.440 (15)	-2.342‐12.092	.17
GLTEQ^e^	97.81 (84.680)	101.00 (93.047)	-0.237 (15)	-31.890‐25.515	.82
PHQ-9^f^	11.88 (4.674)	7.69 (3.860)	4.559 (15)	2.230‐6.145	<.001
GAD-7^g^	9.75 (4.946)	7.31 (4.316)	3.538 (15)	0.969‐3.906	.003
EQ-5D-3L^h^	3.06 (2.932)	2.13 (2.579)	2.611 (15)	0.172‐1.703	.02

a Paired *t* tests were used to compare preintervention and postintervention scores.

b BPI: Brief Pain Inventory.

c PCS: Pain Catastrophizing Scale.

d FABQ: Fear-Avoidance Beliefs Questionnaire.

e GLTEQ: Godin Leisure Time Exercise Questionnaire.

f PHQ-9: Patient Health Questionnaire-9 items.

g GAD-7: Generalized Anxiety Disorder-7 items.

h EQ-5D-3L: EuroQol 5-Dimension 3-Level.

**Table 5.** Session-specific satisfaction scores of each session (N=16).

Session	Description		Understandability		Comprehensibility		Applicability	
	Education	PA^a^	Mean (SD)		Mean (SD)		Mean (SD)	
Session 1	Definition of pain	Warm and balancing	4.06 (0.772)		4.01 (0.771)		4.00 (0.966)	
Session 2	Biopsychosocial aspects of CP^b^	Stretching^c^	4.19 (0.750)		4.19 (0.750)		4.06 (0.998)	
Session 3	Mechanism of CP	Core strength training	4.38 (0.719)		4.37 (0.719)		4.19 (0.981)	
Session 4	Mechanism of CP	Stretching^c^	4.31 (0.793)		4.32 (0.763)		4.13 (1.204)	
Session 5	Pharmacological intervention	Core strength training	4.25 (0.856)		4.25 (0.856)		3.88 (1.360)	
Session 6	Nonpharmacological management	Core strength training	4.38 (0.719)		4.31 (0.732)		4.06 (1.237)	
Session 7	PA	Stretching^c^	4.19 (0.981)		4.19 (0.985)		4.13 (0.957)	
Session 8	Goal setting strategies	Core strength training	4.19 (1.047)		4.22 (1.041)		4.00 (1.211)	

a PA: physical activity.

b CP: chronic pain.

c Stretching for head, neck or shoulder, low back, and knee.

### Predictive Modeling and Key Determinants of Treatment Response

Gradient boosting and random forest models exhibited superior performance compared to other models. Specifically, gradient boosting achieved an AUC of 0.89, *F*_1_-score of 0.82, accuracy of 0.83, precision of 0.82, and recall of 0.81, demonstrating predictive accuracy for pain intensity changes. The random forest model also performed well (AUC=0.87, *F*_1_-score=0.80, accuracy=0.81, precision=0.80, and recall=0.79) (Table 6).

**Figure 2.**
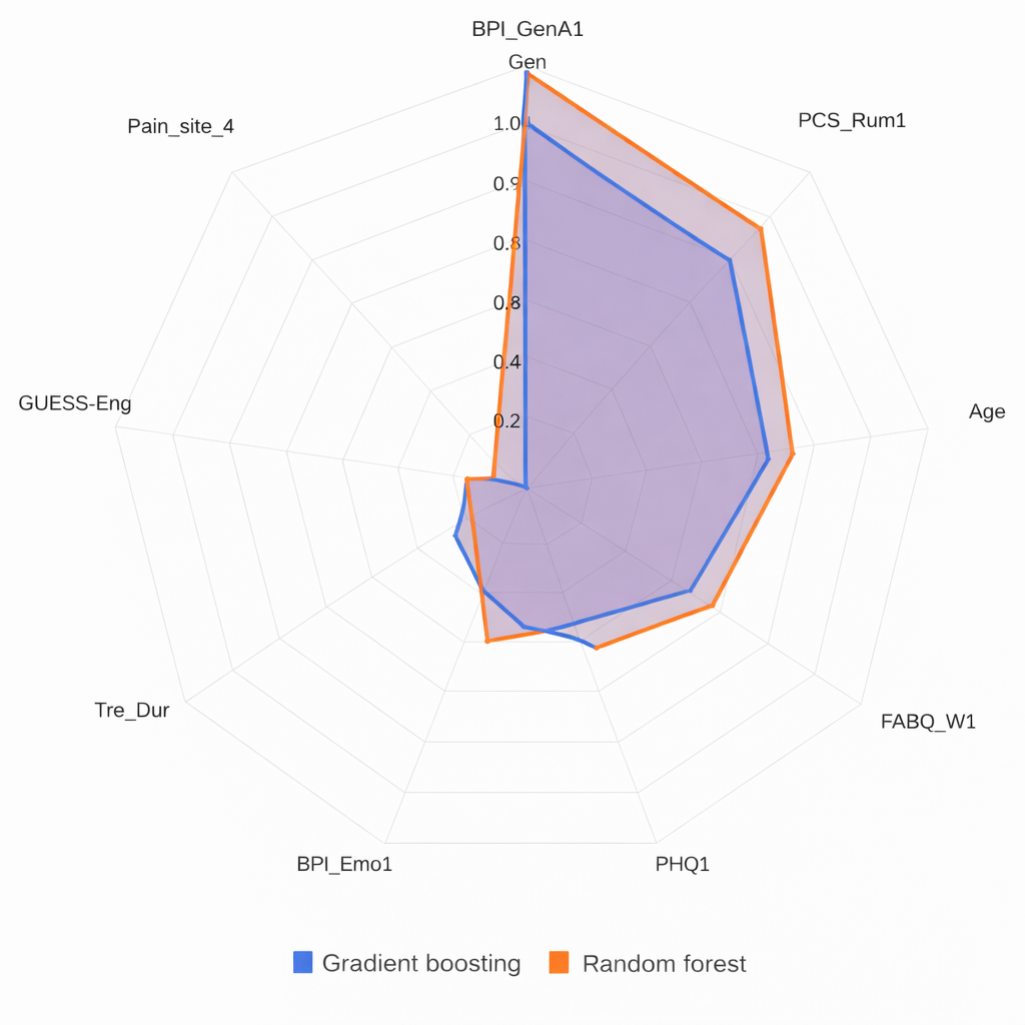
Average absolute Shapley additive explanations values from gradient boosting and random forest models for predicting pain reduction. BPI: Brief Pain Inventory; FABQ: Fear-Avoidance Beliefs Questionnaire; GUESS: Game User Experience Satisfaction Scale; PCS: Pain Catastrophizing Scale; PHQ: Patient Health Questionnaire.

Figures2  3 illustrate SHAP value analysis, identifying the most influential predictors of treatment response. Pain interference with general activity (BPI_GenA1) emerged as the strongest predictor, suggesting that participants with greater pain interference experienced more significant reductions in pain intensity after the GPM intervention. Pain catastrophizing (rumination) and age emerged as the second most significant predictors, with variations in these measures substantially affecting intervention effectiveness. Fear-avoidance belief demonstrated a significantly negative effect on prediction. Features including depression, TD, engrossment in the game (GUESS_Eng), and pain site are also identified as highly influential factors. These patterns suggest that participants with greater pain impact and consistent engagement derive the most benefit from GPM.

**Table 6.** Performance comparison of machine learning models for predicting clinically significant pain reduction (N=16).

Model	AUC^a^	Accuracy	Recall	Specificity	Precision	*F*_1_-score^b^	% positive^c^
Gradient boosting	0.89	0.83	0.81	0.85	0.82	0.82	50%
Random forest	0.87	0.81	0.79	0.83	0.8	0.8	
Logistic regression	0.76	0.72	0.7	0.74	0.71	0.71	
SVM^d^	0.74	0.7	0.68	0.72	0.69	0.69	
Decision tree	0.73	0.69	0.67	0.71	0.68	0.68	

a AUC: area under the curve.

b *F*-score: the harmonic mean of precision and recall.

c % positive: clinical positive outcome (reduction of 30% compared to the baseline in pain intensity).

d SVM: support vector machine.

**Figure 3.**
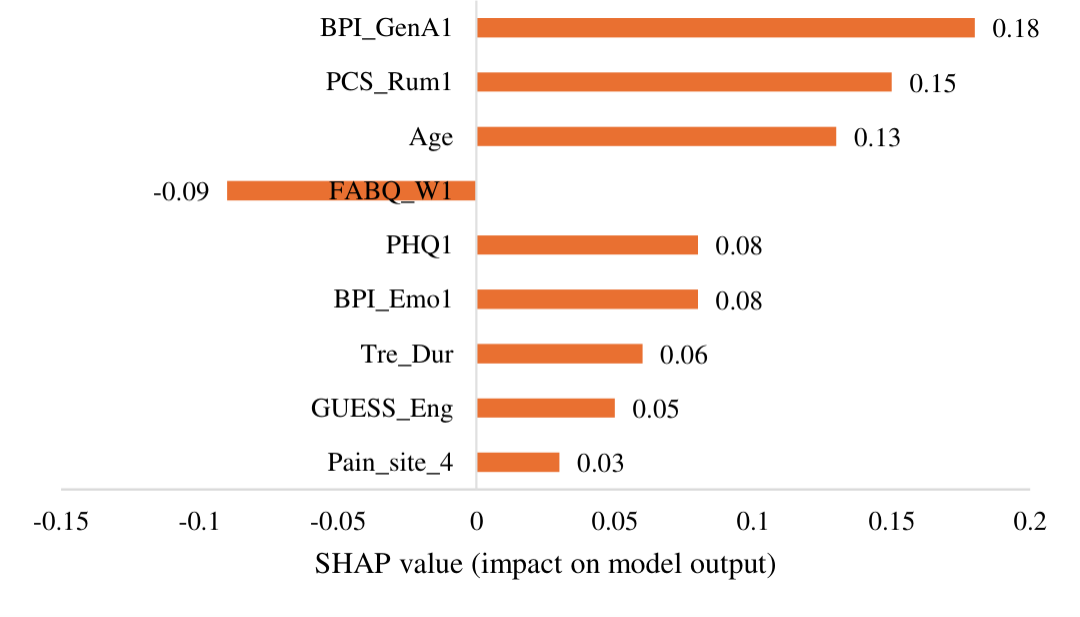
SHAP value for gradient boosting model predicting pain reduction. BPI: Brief Pain Inventory; FABQ: Fear-Avoidance Beliefs Questionnaire; GUESS: Game User Experience Satisfaction Scale; PCS: Pain Catastrophizing Scale; SHAP: Shapley additive explanations.

### Session Level Mechanisms of Clinical Improvement

We conducted further analysis to examine session-level engagement patterns. Table 7 shows LASSO regression results linking SAT to pain improvement. Session 3 and 5 were significant predictors (*P*<.05), indicating that higher satisfaction in these sessions was associated with greater reductions in pain intensity. However, other sessions were assigned near-zero coefficients, suggesting minimal influence on treatment outcomes (Table 7).

**Figure 4.**
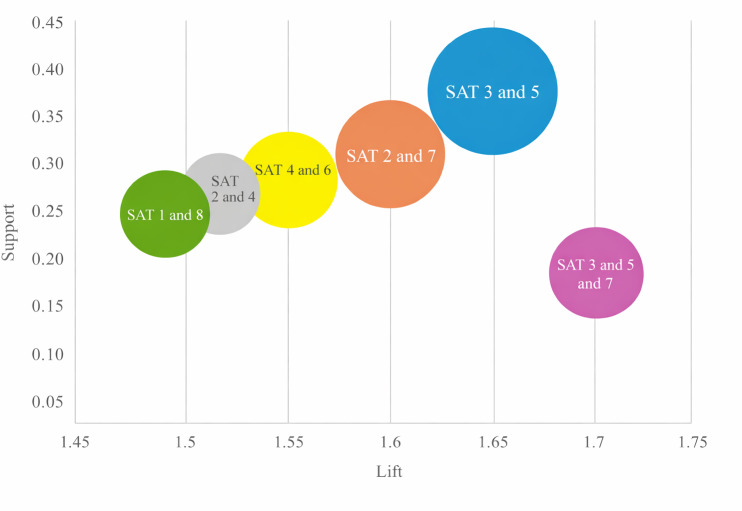
The bubble diagram of the grouping matrix for the 6 association rules. SAT: session satisfaction scores.

Apriori association rule analysis identified effective combinations of session engagement associated with clinical improvement. The combination of high satisfaction in session 3 and session 5 exhibited the strongest association with clinical improvement (support: 0.38, confidence: 0.83, and lift: 1.65), particularly among participants with severe pain. The combination of sessions 4 and 6 was associated with improvement in the mild-to-moderate pain (MMP) subgroup (confidence: 0.78). Additionally, the combination of sessions 3, 5, and 7 had the highest confidence (0.85) and lift (1.70), suggesting a particularly potent effect for participants with severe low back pain (LBP). Other session combinations analysis is provided in Table 8 and Figure 4.

**Table 7.** Least absolute shrinkage and selection operator regression coefficients identifying key session-specific satisfaction scores associated with clinical pain improvement (N=16).

Session	Coefficient	SE	*z* score	*P* value^a^	95% CI
SAT 1^b^	−0.05	0.19	−0.26	.79	−0.42 to 0.32
SAT 2	−0.18	0.16	−1.13	.26	−0.49 to 0.13
SAT 3	−0.45	0.12	−3.75	<.001	−0.68 to −0.22
SAT 4	−0.12	0.17	−0.71	.48	−0.45 to 0.21
SAT 5	−0.32	0.14	−2.29	.02	−0.59 to −0.05
SAT 6	−0.08	0.18	−0.44	.66	−0.43 to 0.27
SAT 7	−0.28	0.15	−1.87	.06	−0.57 to 0.01
SAT 8	−0.03	0.20	−0.15	.88	−0.42 to 0.36

a Statistical significance was determined using the least absolute shrinkage and selection operator regression. Negative coefficients indicate higher satisfaction associated with greater pain reduction.

b SAT: session satisfaction scores.

**Table 8.** Association rule patterns of session satisfaction combinations associated with clinically significant pain improvement by pain subgroup (N=16).

Session combination^a^	Support	Confidence	Lift	Subgroup
High SAT^b^ 3 and 5	0.38	0.83	1.65	Severe pain
High SAT 4 and 6	0.29	0.78	1.55	MMP^c^
High SAT 1 and 8	0.25	0.75	1.50	Other pain
High SAT 2 and 4	0.27	0.77	1.53	N/SP^d^
High SAT 2 and 7	0.31	0.80	1.60	LBP^e^
High SAT 3, 5, and 7	0.19	0.85	1.70	Severe and LBP
Comprehensibility and applicability	0.22	0.81	1.68	Severe pain
High SAT 6 and applicability	0.17	0.78	1.52	MMP
High SAT 3 and comprehensibility	0.18	0.82	1.63	LBP
High SAT 5 and understandability	0.20	0.79	1.58	N/SP

a Data was analyzed by using Apriori association rules to identify frequent item sets of session satisfaction associated with clinical improvement. Higher lift (>1.5) indicates strong positive association between session combination and clinical improvement.

b SAT: session satisfaction scores.

c MMP: mild-to-moderate pain.

d N/SP: neck or shoulder pain.

e LBP: low back pain.

### Time-to-Improvement Analysis Across Subgroups

**Figure 5.**
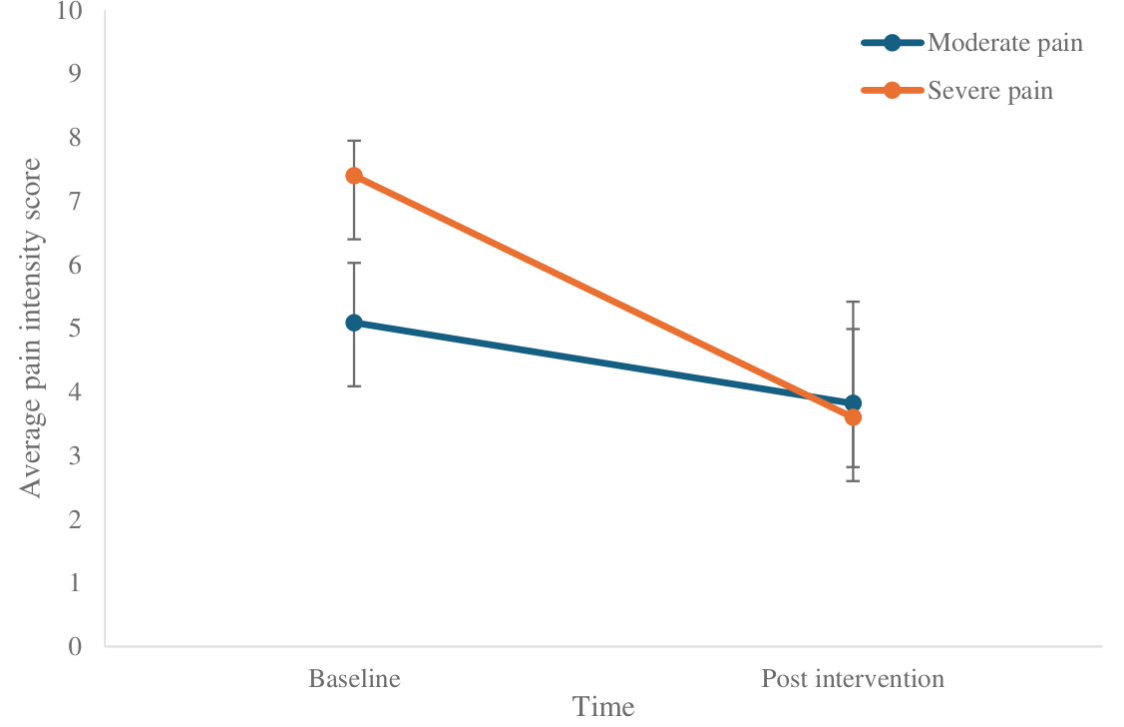
Comparison of Brief Pain Inventory intensity scores for mild-to-moderate pain and severe pain across time.

Table 9 presents within-group effects across different pain subgroups. Significant reductions in pain intensity were observed from baseline to post intervention in the MMP (*t*_10_=3.130, 95% CI 0.367-2.179; *P*=.01), severe pain group (*t*_4_=4.750, 95% CI 1.579, 6.021; *P*=.009), and neck or shoulder pain (N/SP) group (*t*_6_=5.925, 95% CI 1.075-2.925; *P*=.002), indicating that the intervention had a statistically significant effect across multiple subgroups (Figures5  6). These differences indicate that treatment response may depend on pain site and baseline intensity.

**Table 9.** Within-group pre-to-post changes in pain intensity across pain subgroups (N=16).

Group	Participants, n (%)	Baseline	Post intervention	Statistics		
		Mean (SD)	Mean (SD)	*t* test (*df*)	95% CI	*P* value^a^
MMP^b^	11 (68.75)	5.09 (0.94)	3.82 (1.17)	3.130 (10)	0.367 to 2.179	.01
Severe pain	5 (31.25)	7.40 (0.55)	3.60 (1.82)	4.750 (4)	1.579 to 6.021	.009
Other pain	4 (25)	5.75 (2.22)	2.75 (0.96)	2.121 (3)	−1.501 to 7.501	.12
N/SP^c^	7 (43.75)	5.86 (1.07)	3.86 (1.07)	5.925 (6)	1.075 to 2.925	.002
LBP^d^	5 (31.25)	5.80 (1.30)	4.40 (1.67)	1.510 (4)	−1.175 to 3.975	.21

a Paired *t* tests were used to compare preintervention and postintervention scores.

b MMP: mild-to-moderate pain.

c N/SP: neck or shoulder pain.

d LBP: low back pain.

**Figure 6.**
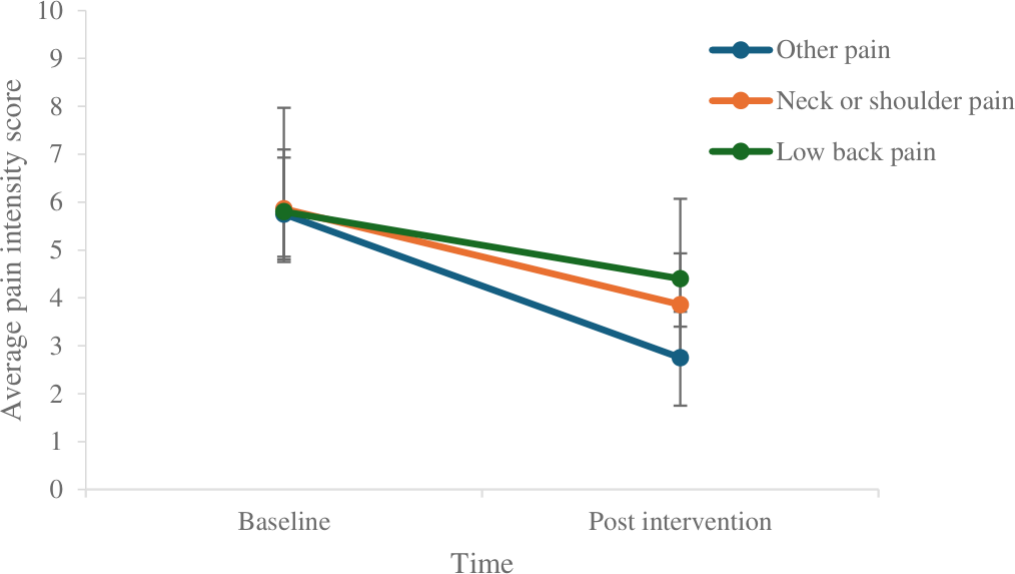
Comparison of Brief Pain Inventory intensity scores for different pain site groups across time.

SHAP values indicated that participants in the severe pain group required longer treatment (≥5.0 wk) for significant benefit, with session 3 identified as the top session-level predictor, while TD dominated overall predictions. An increase to 80% probability of clinically significant improvement benefit from a minimum of 5 weeks in the severe group and 3.6 weeks in the MMP group. LBP benefited after 4.6 weeks and N/SP after 4 weeks, suggesting that dose response varied by condition (Table 10).

**Figure 7.**
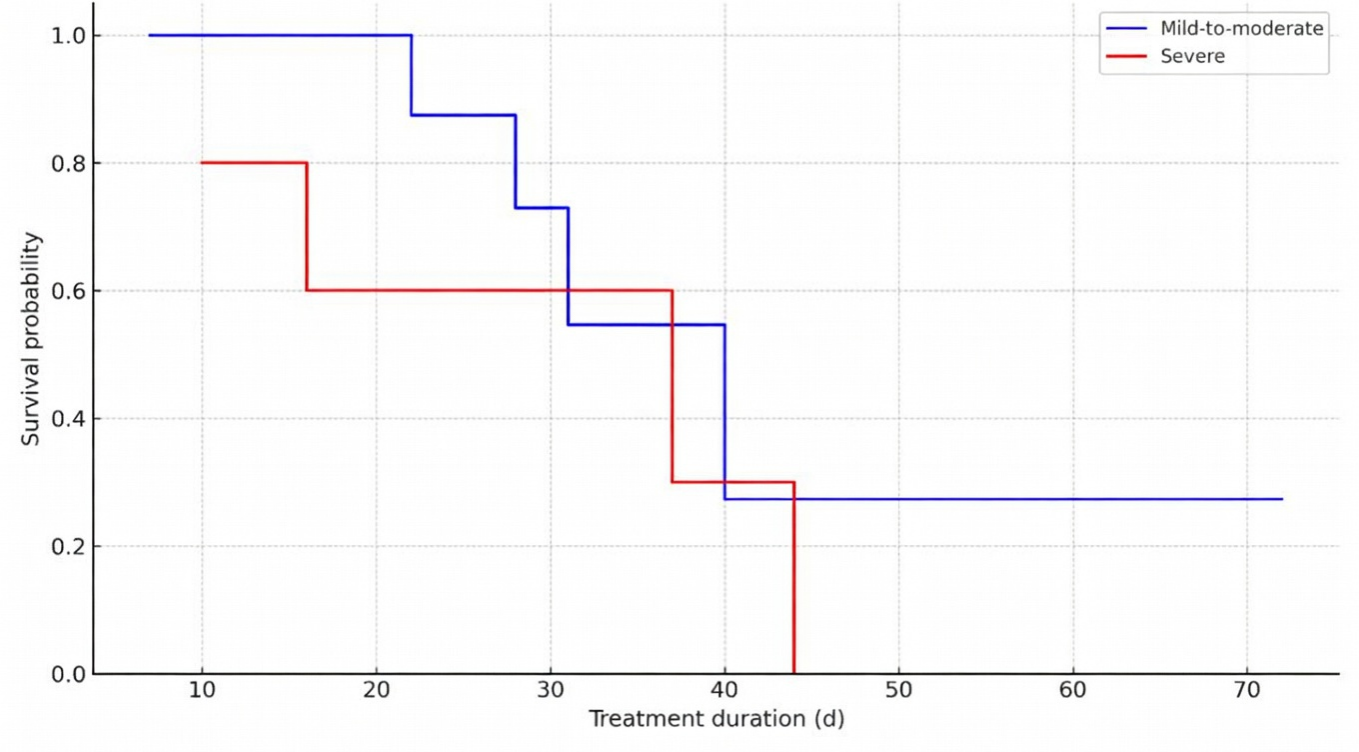
Kaplan-Meier survival curves for time to clinical improvement by pain intensity group.

**Figure 8.**
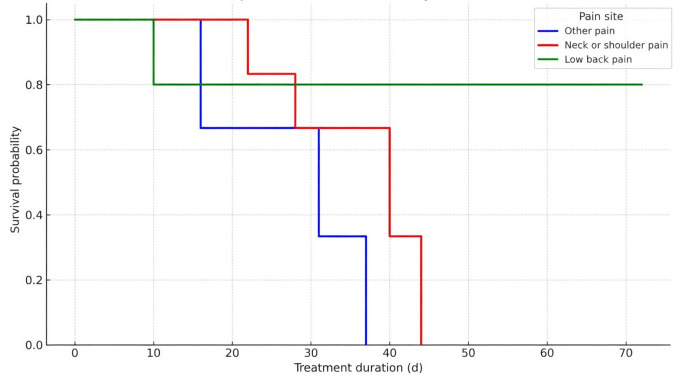
Kaplan-Meier survival curves for time to clinical improvement by pain site group.

Kaplan-Meier survival curves were generated for participants stratified by baseline pain intensity and pain site, corroborating findings with SHAP-based threshold analysis. The median time to improvement was longer for participants with severe pain (5 wk) compared to those with MMP (3.6 wk), and the difference was significant (log-rank *P*=.03) (Table 11, Figure 7). The median time to improvement was 4.6 weeks for LBP, which was longer than N/SP (3.4 wk) and other pain group (4 wk) (Table 10, Figure 8). Bootstrap validation provided support for the stability of these estimates (Table 12). However, these findings require confirmation in larger samples.

**Table 10.** SHAP^a^-informed thresholds for optimal treatment duration by subgroup (N=16).

Group	Optimal TD^b^ (wk)	Probability increase (%)	Key predictors (SHAP values)
Severe pain	≥5.0	+80	SAT^c^ 3 (0.45), TD (0.30)
MMP^d^	≥3.6	+70	SAT 4 (0.28), SAT 6 (0.22)
LBP^e^	≥4.6	+75	SAT 7 (0.35), TD (0.25)
N/SP^f^	≥4.0	+65	SAT 2 (0.40), SAT 4 (0.20)
Other pain	≥3.4	+60	SAT 1 (0.30), SAT 8 (0.25)

a SHAP: Shapley additive explanations.

b TD: treatment duration.

c SAT: session satisfaction scores.

d MMP: mild-to-moderate pain.

e LBP: low back pain.

f N/SP: neck or shoulder pain.

**Table 11.** Kaplan-Meier survival estimates of time to clinically significant improvement by subgroup (N=16).

Group	TD^a^ (wk), median	3-wk PCI^b^ (%)	6-wk PCI (%)	Log-rank *P*
MMP^c^	3.6	50	90	0.03
Severe pain	5	20	70	0.03
LBP^d^	4.6	25	80	0.04
N/SP^e^	4	40	85	0.05
Other pain	3.4	55	75	0.15

a TD: treatment duration.

b PCI: proportion with clinical improvement.

c MMP: mild-to-moderate pain.

d LBP: low back pain.

e N/SP: neck or shoulder pain.

**Table 12.** Bootstrap validation of median time-to-improvement estimates by pain subgroup (N=16).

Group	KM^a^ survival analysis (wk), median	Bootstrap^b^ (wk), median	95% CI
Severe pain	5.0	4.9	4.4-5.4
MMP^c^	3.6	3.7	3.1-4.1
LBP^d^	4.6	4.4	4.0-5.0
N/SP^e^	4.0	3.9	3.4-4.4
Other pain	3.4	3.6	3.0-4.3

a KM: Kaplan-Meier.

b Bootstrap validation, using 1000 iterations, was conducted to assess the robustness and reliability of the median time to clinical improvement.

c MMP: mild-to-moderate pain.

d LBP: low back pain.

e N/SP: neck or shoulder pain.

## Discussion

### Principal Findings

This pilot study examined the preliminary effectiveness of GPM and explored predictive factors of clinically significant improvement using ML models, demonstrating significant effectiveness. The ML model analysis revealed that baseline pain interference, pain catastrophizing, fear-avoidance beliefs, and game engagement were identified as key predictors. The findings also highlight the optimal sessions and intervention duration by pain intensity and site subgroups, suggesting the potential for personalized pathways to achieve optimal outcomes.

### Interpretation

The GPM intervention demonstrated significant improvement in pain and psychological outcomes, supporting that the integration of game elements can effectively address both physical and psychosocial dimensions of CP [9,10]. The superior performance of the gradient boosting classifier in predicting clinically significant pain reduction, consistent with previous studies, suggests that treatment outcomes are multifactorial [37-39]. The strongest predictor was baseline pain interference rather than pain intensity, supported by the Health Belief Model that individuals perceiving higher functional impairment may benefit more from self-management [40]. Furthermore, our findings demonstrated the role of cognitive and affective factors. Rumination of pain catastrophizing was a key predictor, magnifying the negative cognitive loop of pain that is related to disability [41,42]. Similarly, the impact of fear-avoidance beliefs, especially work-related fear-avoidance, suggests that maintaining daily function may be more critical than purely physical rehabilitation [43]. These findings highlight that pain management should primarily address the barriers which are strongly related to daily lives rather than pain intensity score.

Our findings also identified session 3 and session 5 as key contributors to clinical pain recovery. Both of these sessions introduced core strength training, which was more physically demanding [44,45]. Psychoeducational content focusing on pain reconceptualization, including the alarm system and safe analgesic use, may foster self-efficacy by simultaneously providing patients with tangible tools to reduce pain-related fear and misunderstanding [46-49]. The combination of sessions 3, 5, and 7 further suggests that sequencing-targeted exercises with specific educational content may be more effective than a general exercise program. In addition, this combination was most potent for severe LBP, further suggesting that a specific sequence and combination of content are critical. The SHAP-informed modeling and Kaplan-Meier analysis further indicated that individuals with severe pain or LBP required a longer intervention period to achieve a high probability of clinical benefit compared to those with MMP or N/SP. These findings suggest an adaptive intervention strategy that patients with severe LBP could be guided toward a curriculum emphasizing core strength and pain neuroscience education (such as sessions 3 and 5) and encouraged to adhere to at least 5 weeks, while patients with neck or shoulder pain might achieve similar outcomes with a shorter intensive program.

This study established and extended previous studies on digital GPM. First, despite the existing gamified interventions [11,14,50] (Table S2 in Multimedia Appendix 1), they often reported heterogeneous effects and seldom tracked intraintervention engagement behaviors and time-to-improvement thresholds. Our study addresses this by integrating self-tracking function and satisfaction, quiz accuracy recorded functions, allowing us to analyze the high impact intervention components. Second, while recent literature on the application of ML in CP management reveals promise, most studies relied on baseline patient data rather than intraintervention dynamics, lacking behavioral, and engagement metrics [51]. Other studies focused on predictive accuracy including diagnosis or referral need [11,19,51-54], without detailing modeling or treatment engagement, session-by-session behaviors or time-to-improvement. Our study incorporated both baseline and continuously recorded variables into the ML algorithms to offer an adaptive framework for future personalized GPM intervention.

### Limitations

This study has several limitations that should be acknowledged. First, the small sample size limits the statistical power and generalizability of the findings [55]. The risk of overfitting in the ML models is considerable given the high feature-to-sample ratio, although cross-validation was used to mitigate this. These results should therefore be considered preliminary and hypothesis-generating for future larger-scale studies. As the employed participants may exhibit higher motivation to reduce work-related inequities and return to work [56], larger and more diverse samples are needed to confirm the results and understand the intervention’s broader applicability. Additionally, the absence of an external validation dataset limits the assessment of the ML model’s generalizability. Future studies should validate model performance using independent datasets. Moreover, given the high feature-to-sample ratio, there remains a risk of overfitting despite cross-validation. Dimensionality reduction techniques or regularization methods should be considered in future models. Moreover, reliance on self-reported measures, such as pain intensity, interference, and engagement-related behaviors, may introduce subjective bias. Incorporating objective monitoring tools or physiological measures could complement self-report data and enhance data accuracy. In addition, recruitment via social media may introduce selection bias, as individuals with higher digital literacy differ from the broader CP population. Future studies should aim for more inclusive participant recruitment strategies to enhance the generalizability of the findings.

### Implications

While the findings should be interpreted with caution due to the small sample size, this study highlights the potential of gamified interventions and its accompanying ML models in managing CP. The most immediate implications stem from the ML models, which demonstrated potentially predictive accuracy. With a larger sample size, clinicians could use these models as a decision-support tool to personalize pain management strategies, ensuring more effective and tailored treatment plans. To implement the proposed gradient boosting model in clinical practice, the model should be integrated into a digital platform where clinicians input patient data, incorporating data quality check by using valid scale and halt predictions for missing or invalid inputs to ensure reliability. The clinician role would be to accurately administer the required patient-reported outcome measures and interpret the probability of clinical improvement. Furthermore, the preliminary findings underscore the necessity of integrating psychological support within gamified programs. Addressing factors such as pain catastrophizing and fear-avoidance beliefs appears as crucial as targeting physical symptoms for improving outcomes. Finally, the analysis of intraintervention metrics and session-specific effects across multiple pain subgroups offers a foundation for future research, enabling the development of tailored interventions with optimized treatment lengths and content sequencing. Future research should also evaluate the feasibility of GPM through controlled trials with multiple populations.

### Conclusion

In conclusion, this pilot study highlights the potential of leveraging ML models to analyze dynamic data from GPM to inform adaptive and personalized CP management. The study transcends the traditional reliance on static baseline data and overall efficacy assessments of previous studies, instead integrating continuously recorded behavioral metrics to identify key predictors, optimal session combinations, and subgroup-specific TDs. These exploratory findings present a process-oriented, data-driven paradigm that could potentially assist health care providers in customizing intervention content, sequencing, and dosage to enhance adherence in clinical practice. Future larger-scale controlled trials are necessary to validate these exploratory models and convert them into clinical decision-support systems that could improve patient involvement and outcomes.

## Supplementary material

10.2196/78823 Multimedia Appendix 1 Detailed preintervention and postintervention comparisons of pain and psychological outcomes.

10.2196/78823 Checklist 1 TRIPOD-AI checklist.

## Abbreviations

AI: artificial intelligence
AUC: area under the curve
CP: chronic pain
GPM: gamified pain management
GUESS: Game User Experience Satisfaction Scale
LASSO: least absolute shrinkage and selection operator
LBP: low back pain
ML: machine learning
MMP: mild-to-moderate pain
N/SP: neck or shoulder pain
PHQ-9: Patient Health Questionnaire-9 items
SAT: session satisfaction scores
SDT: self-determination theory
SHAP: Shapley additive explanations
TD: treatment duration
TRIPOD-AI: Transparent Reporting of a Multivariable Prediction Model for Individual Prognosis or Diagnosis-AI
