# Supplementary material for: A Gamified Pain Management Intervention for Adults With Chronic Pain in Mainland China: Single-Arm Pre-Post Pilot Study With Machine Learning Predictive Modeling
Source: JMIR Form Res. 2026 Apr 7;10:e78823. doi: 10.2196/78823 (PMC13055939; doi:10.2196/78823)
Supplement: Multimedia Appendix 1 [file formative-v10-e78823-s001.docx]

**Supplementary Table 1. Detailed pre- and post-intervention comparison of pain and psychological outcomes (n=16)**

|  | **Baseline** | | **Post intervention** | |  |  |  |  |
| --- | --- | --- | --- | --- | --- | --- | --- | --- |
|  | **M** | **SD** | **M** | **SD** | **t** | **95% CI** | ***df*** | ***P*** ^a^ |
| BPI intensity | 5.81 | 1.377 | 3.75 | 1.342 | 4.392 | 1.061-3.064 | 15 | .001 |
| BPI interference | 38.31 | 12.408 | 25.63 | 12.387 | 5.971 | 8.159-17.216 | 15 | <.001 |
| PCS | 35.81 | 8.796 | 27.31 | 13.200 | 3.190 | 2.821-14.179 | 15 | .006 |
| PCS rumination | 15.38 | 5.188 | 11.19 | 6.221 | 3.226 | 1.421-6.954 | 15 | .006 |
| PCS magnificence | 8.31 | 2.301 | 6.50 | 3.521 | 2.404 | 0.205-3.420 | 15 | .03 |
| PCS helplessness | 12.13 | 2.705 | 9.63 | 4.319 | 2.510 | 0.377-4.623 | 15 | .02 |
| FABQ | 58.69 | 13.189 | 53.81 | 16.586 | 1.440 | -2.342-12.092 | 15 | .17 |
| FABQ PA | 17.69 | 3.701 | 17.31 | 3.860 | 0.441 | -1.439-2.189 | 15 | .67 |
| FABQ work | 23.56 | 5.853 | 20.69 | 7.382 | 1.961 | -0.250-6.000 | 15 | .07 |
| GLTEQ | 97.81 | 84.680 | 101.00 | 93.047 | -0.237 | -31.890-25.515 | 15 | .82 |
| Severe exercise | 4.00 | 5.785 | 4.38 | 6.449 | -0.411 | -2.320-1.570 | 15 | .69 |
| Moderate exercise | 6.25 | 5.053 | 6.25 | 5.859 | 0.000 | -1.876-1.876 | 15 | <.001 |
| Mild exercise | 10.19 | 7.450 | 10.13 | 5.807 | 0.052 | -2.515-2.640 | 15 | .96 |
| PHQ-9 | 11.88 | 4.674 | 7.69 | 3.860 | 4.559 | 2.230-6.145 | 15 | <.001 |
| GAD-7 | 9.75 | 4.946 | 7.31 | 4.316 | 3.538 | 0.969-3.906 | 15 | .003 |
| EQ-5D-3L | 3.06 | 2.932 | 2.13 | 2.579 | 2.611 | 0.172-1.703 | 15 | .02 |
| EQ-5D-3L -Mobility | .94 | 1.063 | 0.56 | 0.892 | 2.087 | -0.008-0.758 | 15 | .05 |
| EQ-5D-3L -Self care | .69 | 1.195 | 0.63 | 1.088 | 0.293 | -0.393-0.518 | 15 | .77 |
| EQ-5D-3L -Usual activity | 1.44 | 1.209 | 0.94 | 1.124 | 2.739 | 0.111-0.889 | 15 | .02 |

**Supplementary Table 2. Summary of recent digital chronic pain management studies**

| **Study** | **Modality** | **Technologies** |
| --- | --- | --- |
| **Gamification** | | |
| Harvie et al., 2024 [14] | VR headsets with avatar guided movement training | Game mechanics: avatar, feedback, tutorials |
| Sun et al., 2024 [50] | Somatosensory exercise game using smartphone motion control | Game mechanics: points, reward |
| Vermeir et al., 2025 [11] | Web-based attentional bias modification training | Game mechanics: feedback, leaderboard, points |
| **ML models** | | |
| Amorim et al., 2021 [19] | ML on rehabilitation sensor data | SVM and Decision Tree using baseline clinical and sensor features for pain intensity classification |
| \| Fundoiano-Hershcovitz et al., 2023 [51] \| \| --- \| | Digital pain-management platform with adaptive ML feedback | Adaptive ensemble model using real-time pain scores and demographics to personalize treatment recommendations |
| \| Lian et al., 2025 [52] \| \| --- \| | Gene based pain outcome prediction | Random Forest and SVM on genomic features for treatment biological predictors |
| Areias et al., 2024 [53] | Remote care program with ML prediction tool including exercise and education | XGBoost using baseline and usage data at 4 and 8 weeks to pain response prediction |
| Skoric et al., 2025 [54] | mhealth App data including daily reflection and the pain record | Gradient Boosting and Logistic Regression on usage patterns for functional improvement prediction |

VR: virtual reality; ML: machine learning; SVM: support vector machines; XGBoost: EXtreme Gradient Boosting.
